# Nasal High Flow at 25 L/min or Expiratory Resistive Load Do Not Improve Regional Lung Function in Patients With COPD: A Functional CT Imaging Study

**DOI:** 10.3389/fphys.2021.683316

**Published:** 2021-06-10

**Authors:** Julien G. Cohen, Ludovic Broche, Mohammed Machichi, Gilbert R. Ferretti, Renaud Tamisier, Jean-Louis Pépin, Sam Bayat

**Affiliations:** ^1^Department of Radiology, Grenoble University Hospital, Grenoble, France; ^2^Department of Imaging, Neuchatel Hospital Network (RHNE), Neuchatel, Switzerland; ^3^European Synchrotron Radiation Facility, Grenoble, France; ^4^STROBE Laboratory, INSERM UA7, Grenoble-Alps University, Grenoble, France; ^5^HP2 Laboratory, INSERM U1042, Grenoble-Alps University, Grenoble, France; ^6^Department of Pulmonology and Physiology, Grenoble University Hospital, Grenoble, France

**Keywords:** nasal high flow cannula, expiratory resistive loading, COPD, computed tomography, image processing

## Abstract

**Background:**

Nasal high flow (NHF) is a non-invasive breathing therapy that is based on the delivery via a large-caliber nasal cannula of heated and humidified air at flow rates that exceed peak inspiratory flow. It is thought that positive airway pressure generated by NHF can help reduce gas trapping and improve regional lung ventilation. There are no data to confirm this hypothesis at flow rates applicable in stable chronic obstructive pulmonary disease (COPD) patients.

**Methods:**

In this study, we used non-rigid registration of computed tomography (CT) images acquired at maximal expiration and inspiration to compute regional lung attenuation changes (ΔHU), and lung displacement (LD), indices of regional lung ventilation. Parametric response maps (Galban et al., 2012) were also computed in each experimental condition. Eight COPD patients were assessed at baseline (BL) and after 5 min of NHF and expiratory resistive loading (ERL).

**Results:**

ΔHU was: BL (median, IQR): 85 (67.2, 102.8); NHF: 90.7 (57.4, 97.6); ERL: 74.6 (46.4, 89.6) HU (*p* = 0.531); and LD: 27.8 (22.3, 39.3); 17.6 (15.4, 27.9); and 20.4 (16.6, 23.6) mm (*p* = 0.120) in the 3 conditions, respectively. No significant difference in trapping was observed. Respiratory rate significantly decreased with both treatments [BL: 17.3 (16.4, 18.9); NHF: 13.7; ERL: 11.4 (9.6, 13.2) bpm; and *p* < 0.001].

**Conclusion:**

Neither NHF at 25 L/min nor ERL significantly improved the regional lung ventilation of stable COPD patients with gas trapping, based on functional lung CT imaging. Further study including more subjects is needed to assess the potential effect of NHF on regional lung function at higher flow rates.

**Clinical Trial Registration:**

www.clinicaltrials.gov/under, identifier NCT03821311.

## Introduction

Nasal high flow (NHF) is a non-invasive breathing therapy that is based on the delivery via a large-caliber nasal cannula, of heated and humidified air at flow rates that exceed peak inspiratory flow (Drake, 2018). The therapy is used for a variety of conditions including chronic obstructive pulmonary disease (COPD; Ashraf-Kashani and Kumar, 2017). The device consists of an air/oxygen blender connected to a nasal cannula, through a heated and humidified inspiratory circuit. It delivers a fraction of inspired oxygen (FiO2) from 21 to 100%, with a flow rate up to 60 L⋅min^–1^. With this device, FiO2 can be adjusted independently of the flow rate. NHF is increasingly considered as a supportive therapy in critically ill patients with acute respiratory failure, as an alternative to standard oxygen therapy and non-invasive ventilation, including post-operative respiratory failure, or during intubation of patients with mild-to-moderate hypoxemia (Curley et al., 2015). However, there is limited clinical data on the effectiveness of NHF in patients with stable COPD. Moreover, the physiological mechanisms of action of NHF are not fully understood.

Our working hypothesis was that the positive airway pressure generated by NHF particularly during expiration, can help maintain transbronchial pressure and small peripheral airway patency, thereby reducing gas trapping. This mechanism somewhat resembles pursed-lips breathing (PLB), a behavior which is thought to have a similar benefit in severe COPD patients (Mueller et al., 1970). Although small airways cannot be directly imaged, gas trapping and indices of regional lung function can be quantified based on attenuation analysis of registered computed tomography (CT) images obtained at high and low lung volumes. Moreover, indices of regional lung ventilation and motion measured using this novel non-invasive approach which does not rely on exogenous contrast media, have been proposed as biomarkers of functional small airway obstruction (Galban et al., 2012; Chae et al., 2020).

The goal of this study was to assess gas trapping and indices of regional lung function using CT image registration analysis in patients with COPD at baseline (BL) and under NHF at 25 L/min. Because the beneficial effect of PLB in severe COPD patients has a similar hypothetical mechanism to NHF, we also assessed expiratory resistive loading (ERL) to produce a controlled amount of positive pressure only during expiration, with a positive expiratory pressure (PEP) mask, thus mimicking PLB.

## Materials and Methods

### Ethics and Consent

Patients included in this study were part of a COPD cohort of 77 patients attending Grenoble University Hospital (Grenoble, France). This study was performed in accordance with the Declaration of Helsinki. The study was approved by the Comité de Protection des Personnes, Nord Ouest – approval: 2018-A00363-52. Trial registration: clinicaltrials.gov, NCT03821311. Registered 29 January 2019, clinicaltrials.gov/ct2/show/record/NCT03821311. Participant registration took place from Jan-2019 to Dec-2019. All adult participants provided written informed consent to participate in this study.

### Patients

Eight patients with COPD, aged > 18 years, followed up at the Grenoble University Hospital outpatient pulmonology clinic were included in the cohort. Patients with evolving cancer, pregnancy or subject to an exclusion period in another investigation were not included in the study. Of the 77 patients initially included, 8 subsequent patients having accepted to participate in this ancillary study were enrolled.

### Study Protocol

Following BL inspiratory and expiratory CT acquisition, image acquisition was repeated at both lung volumes after 5 min and during NHF (AIRVO-2, Fisher & Paykel, Auckland, New Zealand) using a medium-sized nasal cannula (Optiflow, Fisher & Paykel, Auckland, New Zealand), and after 5 min and during ERL, using a TheraPEP system (Smiths Medical, Portex, United Kingdom) set to level 6, connected to a face mask (Vitera, Fisher & Paykel, Auckland, New Zealand). The order of NHF and ERL treatments was randomized using a random choice generator in Microsoft Excel. Patients were instructed to keep their mouths closed during NHF. 5 to 10 min of washout were allowed between each treatment. Respiratory rate was monitored using a respiratory inductance plethysmography (RIP) belt connected to an analog-digital interface (Powerlab 16/35, ADInstruments, Oxford, United Kingdom). The data were sampled at 1 kHz and recorded on a laptop computer. Lung function parameters were measured using spirometry (FEV1, FVC) with the Global Lung Initiative reference values (Quanjer et al., 2012) and body plethysmography (TLC, RV) was performed using a Medisoft body box (MGC Diagnostics, MN, United States), in accordance with the American Thoracic Society – European Respiratory Society technical recommendations (Graham et al., 2019).

### CT Imaging and Image Processing

Chest CT was performed at the Grenoble University Hospital Department of Radiology, with a 256-slice scanner (GE Revolution CT, GE Medical Systems, Milwaukee, United States) with the following settings: 120 kV, tube current modulation and collimation width of 0.625 mm. Images were acquired upon breath-hold at both full inspiration and full expiration. The patients were instructed by the technician prior to, and coached during image acquisition. Images were reconstructed with a standard convolution kernel. Average dose-length product was 262 ± 47 mGy⋅cm.

**FIGURE 1:**
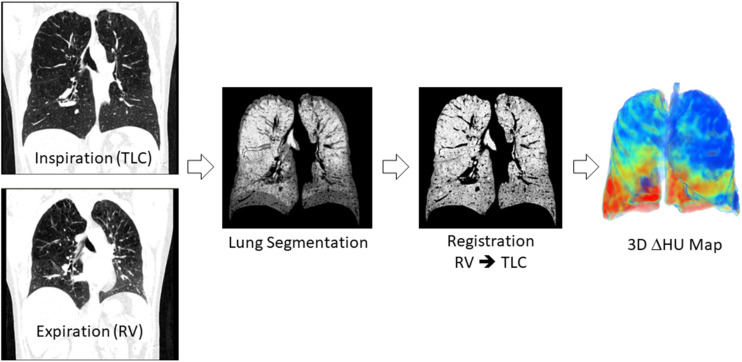
Image processing workflow. Inspiratory and expiratory CT images were segmented using a region growing algorithm. The segmented expiratory image is warped using elastic image registration software to match the inspiratory image. The indices of regional lung ventilation are computed based on the registered and inspiratory images.

Details of the image processing methodology are explained in the online supplement. The image processing workflow is shown in Figure 1. Briefly, images were processed with the python programming language (Python Software Foundation; Python Language Reference, version 2.7), running on a desktop computer (CPU: Intel Xenon @2.4 GHz × 16, 126 GB of RAM and NVIDIA Quadro K5000 GPU). Segmentation of the aerated lung tissue from the CT images was performed using an iterative region growing algorithm (Adams and Bischof, 1994). Within the segmented lung, an aerated voxel was defined by a lung tissue density lower than the median of the density distribution plus two standard deviations: μ + 2σ. An elastic 3D registration method was used to compute motion and deformation between inspiratory and expiratory lung images (Yoo et al., 2002; Kybic and Unser, 2003).

For each voxel of the resulting registered image, the change in attenuation (ΔHU) between end-expiration and end-inspiration was computed for each image voxel. This parameter was the primary outcome of the study. Scattering of regional attenuation change was expressed as the coefficient of variation; CV-ΔHU: ΔHU_SD_/ΔHU_mean_. A regional lung displacement (LD) vector was computed for each voxel of the registered image. The modulus of this vector was used to compute the local LD between expiration and inspiration. The average value for both lungs was defined as the mean Lung Displacement (LD, mm). The inhomogeneity of LD was assessed as the coefficient of variation; CV-LD: LD_SD_/LD_mean_. Parametric response maps were computed based on Galban et al. (2012). These consist in classifying the lung voxels into 4 categories based on attenuation in registered inspiratory and expiratory images. Normal lung voxels were defined by an attenuation > −950 HU at total lung capacity (TLC) and >−856 HU at residual volume (RV) in the expiratory image. Trapping was defined by an attenuation > −950 HU at TLC and <−856 HU at RV. Emphysema was defined by an attenuation < −950 HU at TLC and <−856 HU at RV. Lung voxels with an attenuation <−950 HU at TLC and >−856 HU at RV were categorized as: “emptying emphysema.” These parameters were expressed as a percentage of the corresponding inspiratory volume within each condition.

### Statistical Analysis

Data are presented as median (interquartile range: Q1, Q3). Friedman’s one-way repeated-measures ANOVA on ranks with a Student-Newman–Keuls *post hoc* multiple comparisons procedure was used to test differences between study conditions: Baseline, NHF, and ERL. Pearson correlation was used to assess the relation between TLC, RV and corresponding BL inspiratory and expiratory CT volumes. A *p* < 0.05 was considered as significant. All statistical analyses were performed with Sigmaplot V.13 software (Systat, Berkshire, United Kingdom).

## Results

Baseline patient characteristics are presented in Table 1. One subject had a body mass index > 30. None of the patients were hypercapnic. All patients had a significant degree of gas trapping with an elevated RV. Five patients were Global Initiative for Chronic Obstructive Lung Disease (GOLD) stage 1 (62.5%); 2 patients stage 2 (25%), and one 1 patient stage 3 (12.5%). Baseline inspiratory CT volume significantly correlated with TLC (*R* = 0.89, *p* < 0.01) with a mean bias (plethysmographic TLC – inspiratory CT volume) of -0.66 L. Expiratory CT volume correlated with RV (*R* = 0.71, *p* < 0.05) with and average bias (plethysmographic RV – expiratory CT volume) of 0.31 L.

**TABLE 1:** Subject characteristics.

*n* (F)	8 (3)
Age (year)	62.8 (59.9, 64.1)
Height (cm)	173 (167.5, 175.3)
Weight (kg)	70 (61.5, 77.5)
BMI	24.5 (22.4, 25)
FEV1 (%Pred)	76.2 (55.9, 82.6)
FVC (%Pred)	98.9 (94, 106.1)
FEV1/FVC (%)	57.9 (47.5, 61.7)
TLC (%Pred)	117.1 (113.3, 133.1)
RV (%Pred)	173.7 (146.2, 184.9)
PO_2_ (kPa)	10.2 (9.6, 10.6)
PCO_2_ (kPa)	4.5 (4.5, 4.8)
pH	7.4 (7.4, 7.4)
HCO3^–^ (mmol/L)	23.1 (21.8, 23.9)

*Data are median (Q1,Q3); F, female; BMI, body mass index; FEV1, forced expiratory volume in 1 s; %Pred, percent predicted value; FVC, forced vital capacity; TLC, total lung capacity; and RV, residual volume.*

**FIGURE 2:**
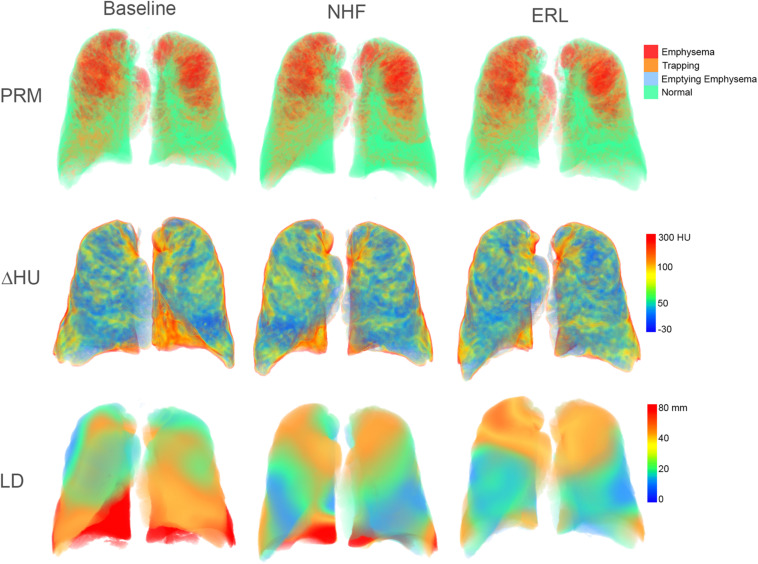
3D rendering of computed regional lung function indices in a sample COPD patient. PRM, parametric response maps; ΔHU, regional attenuation changes from maximal expiration to maximal inspiration; LD, lung displacement; NHF, nasal high flow; and ERL, expiratory resistive loading.

Sample 3D renderings of parametric maps of the various lung functional clusters (Normal; Trapping; Emphysema; and Emptying Emphysema), ΔHU and LD in a representative patient are shown in Figure 2. No significant differences were observed between BL, NHF, and ERL conditions in the lung functional clusters ΔHU, or LD.

Averaged CT image-derived data are presented in Table 2. The volume changes from maximal expiration to maximal inspiration in the CT images tended to decrease with NHF and more so with ERL, however, these changes did not reach statistical significance (*p* > 0.05). The fractional volume of functional clusters defined based on expiratory-inspiratory attenuation change were not significantly different between BL, NHF and ERL (*p* > 0.05). Regarding the other CT registration-based parameters, no significant differences were observed between the experimental conditions.

**TABLE 2:** CT registration-based regional lung function data.

	*BL*	*NHF*	*ERL*	*p*
Volume, expiration [L]	3.6 (3.2, 4.4)	3.9 (3.6, 4.2)	3.8 (3.5, 4.4)	0.236
Volume, inspiration [L]	6.7 (6, 7.3)	6.9 (5.5, 7.5)	6.0 (5.3, 7.1)	0.149
ΔVolume [L]	2.8 (2.2, 3.1)	2.3 (1.9, 2.9)	2.1 (1.8, 2.4)	0.236
RR (bpm)	17.3 (16.4, 18.9)	13.7 (10, 15)*	11.4 (9.6, 13.2)*^§^	<0.001
ΔRIP (mV)	16.7 (12.4, 17.5)	17.6 (8.5, 20.3)	15.4 (13.7, 24.5)	0.566
**PRM clusters:**				
Normal [%] (Green)	68.7 (43.4, 78.3)	69.9 (38.3, 78.9)	62.5 (37.5, 78.6)	0.654
Emptying emphysema [%] (Blue)	1.5 (0.9, 2.6)	0.9 (0.6, 2)	0.8 (0.4, 2.1)	0.236
Trapping [%] (Orange)	22.6 (19.6, 44.7)	22.1 (19.8, 47.7)	29.2 (19.7, 49.9)	0.967
Emphysema [%] (Red)	4.3 (0.8, 12.3)	4.6 (0.4, 12.5)	2.8 (0.7, 13)	0.236
ΔHU [HU]	85 (67.2, 102.8)	90.7 (57.4, 97.6)	74.6 (46.4, 89.6)	0.531
CV−ΔHU	0.9 (0.7, 1.0)	0.9 (0.7, 1.0)	0.9 (0.8, 1.2)	0.531
LD [mm]	27.8 (22.3, 39.3)	17.6 (15.4, 27.9)	20.4 (16.6, 23.6)	0.120
CV−LD	0.4 (0.4, 0.5)	0.5 (0.4, 0.5)	0.5 (0.4, 0.5)	1.00

Data are median (Q1,Q3); BL, Baseline; NHF, nasal high flow cannula at 25 L/min; ERL, expiratory resistive loading; RR, respiratory rate; ΔVolume, Volume change between the expiratory and inspiratory image; ΔRIP, tidal change in respiratory impedance plethysmography signal in millivolts; ΔHU, mean regional lung attenuation change in Hounsfield units; CV−ΔHU, coefficient of variation of ΔHU; LD, mean regional lung displacement in mm; and CV−LD, coefficient of variation of LD.

* *p* < 0.05 vs. BL and ^§^*p* < 0.05 vs. NHF.

**FIGURE 3:**
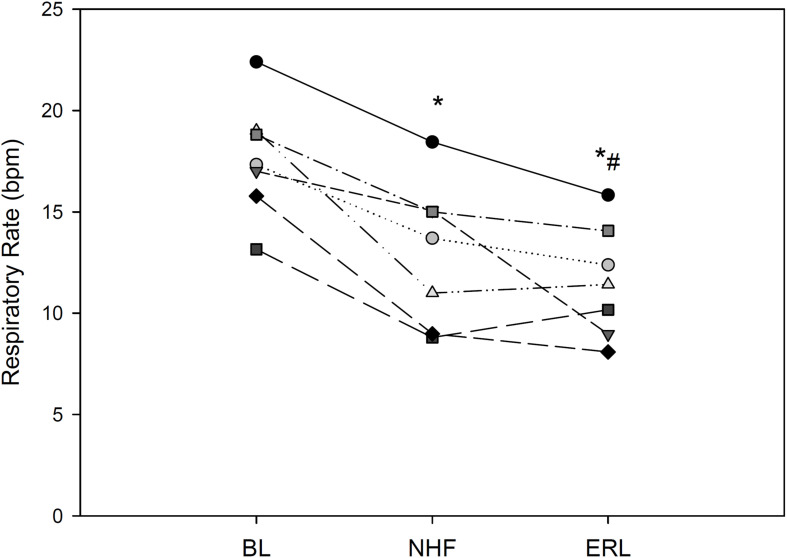
Respiratory rate in each of 8 COPD patients; BL, baseline; NHF, nasal high flow cannula; and ERL, expiratory resistive loading. *p < 0.05 vs. BL, ^#^*p* < 0.05 vs. NHF.

Respiratory rate is shown in Figure 3. Respiratory rate significantly decreased with NHF and further so with ERL [BL: 17.3 (16.4, 18.9); NHF: 13.7; ERL: 11.4 (9.6, 13.2) bpm; *p* < 0.001]. The amplitude of the RIP signal was not significantly different during NHF or ERL despite a tendency to increase with the latter condition (*p* > 0.05, Table 2).

## Discussion

Nasal high flow is increasingly used both within and outside the intensive care setting (Jackson et al., 2021). It is therefore important to better understand its physiological effects on the respiratory system. The goal of this study was to assess gas trapping and indices of regional lung function using CT image registration analysis in order to investigate the short-term effects of NHF in patients with stable COPD, under circumstances different than acute settings such as respiratory failure (Frat et al., 2015; Jones et al., 2016). We further compared NHF to ERL. Our main findings were that NHF reduced respiratory rate and tended to reduce lung volume change from maximal expiration to maximal inspiration, but did not have a significant effect on gas trapping or the regional change in lung attenuation, an index of regional ventilation.

The assessment of traditional respiratory mechanical parameters in patients on NHF is challenging due to the technical difficulty of making measurements at the mouth, because of the high gas flow in the upper airways. It is currently thought that the mechanisms through which NHF improves respiratory function and decreases dyspnea and the work of breathing include: (1) generation of a higher flow rate compared to other oxygen delivery systems, exceeding the patient’s peak inspiratory flow rate, which allows maintaining FiO_2_ relatively constant by reducing the entrainment of room air; (2) washout of CO_2_ from the anatomic dead space, allowing for an improved efficiency of gas exchange; (3) The high gas flow delivered with NHF, although through an open circuit, creates moderate positive nasopharyngeal pressures due to both upper airway resistance and turbulent flow regime, which could contribute to a reduced inspiratory upper airway resistance; and (4) Previously, studies assessing alveolar aeration based on electrical impedance tomography (EIT) have suggested that small positive pressures generated by NHF can contribute to both an increase in lung volume and alveolar recruitment (Corley et al., 2011; Riera et al., 2013), although at higher flow rates than in the present study. However, EIT is not a morphological imaging technique. It is therefore unclear how exactly positive pressure generated by NHF acts on the lung periphery to reduce the work of breathing.

In COPD patients, loss of lung parenchymal tethering causes small airways to collapse early on during expiration, resulting in dynamic compression of the airways, flow limitation, and gas trapping (Takishima et al., 1967). This phenomenon has important implications with respect to mechanical efficiency, the sensation of dyspnea, and exercise limitation in these patients (O’Donnell et al., 1987). Pursed-lips breathing (PLB) is a technique whereby exhalation is performed through a resistance created by constriction of the lips (Mueller et al., 1970). Although the breathing maneuver is often spontaneously adopted by COPD patients, it is also routinely taught as a breathing-retraining exercise in pulmonary rehabilitation programs because it is thought to alleviate dyspnea (Spahija et al., 2005). However, the level of positive pressure induced by PLB is difficult to standardize. Alternatively, ERL can be used in order to produce a graded and controlled amount of positive pressure, only during expiration, thus mimicking PLB (O’Donnell et al., 1987).

Previous studies have measured modest positive airway pressures during NHF therapy, with a mean tracheal pressure of about 2 cmH_2_O at 45 L/min (Chanques et al., 2013) and 3.3 at 50 L/min (Parke et al., 2011), with the same device as ours and with a closed mouth. Because the positive pressure generated by NHF depends on the resistance of the air leak at the nares, it has been recently shown that a snug fit between the nasal prongs and the nares produces higher levels of upper airway positive pressure, using a bench top physical model (Pinkham and Tatkov, 2020). However, even with a larger caliber nasal prong and a snug fit, the generated positive pressure is on the order of 3 cmH_2_O at 25 L/min (Pinkham and Tatkov, 2020). In this study, all patients had medium-sized nasal prongs during NHF therapy. Although upper airway pressure was not measured in this pilot study, it can be expected that the modest positive pressures generated by a flow rate of 25 L/min were insufficient to produce a measurable effect on small airway closure, gas trapping upon expiration, or lung recruitment.

The reduction in respiratory rate observed in this study is in agreement with several previous studies that have described significant decreases in breathing rate and changes in the pattern of breathing during NHF (Corley et al., 2011; Nilius et al., 2013). Our data show that this reduction in respiratory rate was not explained by an improvement in regional lung function in these stable COPD patients, a mechanism often proposed in the literature (Ashraf-Kashani and Kumar, 2017; Pisani and Vega, 2017; Drake, 2018) which has been attributed to PEP induced by NHF. Indeed, an improvement in regional lung ventilation would be expected to increase the change in lung attenuation (ΔHU) as well the local LD, between expiration and inspiration. However, our data show that neither ΔHU nor LD was increased. Other mechanisms such as the washout of CO_2_ from the anatomic dead space, or a drop in upper airway inspiratory resistance may have contributed to the reduction in the work of breathing and respiratory drive with NHF, leading to a reduced respiratory rate. Alternatively, a neural reflex mechanism may be involved. Previously, McBride and Whitelaw demonstrated that circulating air through the nose and mouth of awake healthy subjects at flow rates in the range of tidal breathing, reproducibly inhibited the rate of inspiratory muscle contractions during imposed apnea (McBride and Whitelaw, 1981). The effect was temperature and flow-dependent, allowed longer apnea times when flow was applied, and was abolished by local anesthesia of the nose and pharynx. The authors suggested that upper airway thermal or mechanical receptors through trigeminal, glossopharyngeal and vagally mediated pathways may be involved and that this reflex mechanism could act as a negative feedback to stabilize the breathing pattern. However, we cannot confirm these mechanisms based on the findings of the present study, that focused on regional lung function. We observed similar changes in respiratory rate with ERL. These findings are also in agreement with previous data in the literature (Spahija and Grassino, 1996), although the involved mechanisms may be different from that of NHF (Milic-Emili and Zin, 2011).

The present study had several limitations. Because the standard deviation of the outcome parameters was not known beforehand, a formal sample size estimation was impossible. The small number of enrolled subjects reduced the statistical power. Considering the scattering of the trapped fraction data, inclusion of 8 subjects allows detecting a 20% decrease in trapping with a power of 80% and an α = 0.05. Therefore, we cannot exclude that NHF at 25 L/min may have induced subtler changes in regional lung function in COPD patients who presented significant gas trapping. Only a single flow rate was tested in order to limit radiation exposure due to CT imaging. We chose to investigate a flow rate which would be applicable to stable COPD patients without exacerbation or acute respiratory failure. For the same reason as above, a single nasal prong size was assessed. Therefore, the findings of this study, do not exclude the possibility that regional lung function could be improved with larger nasal prong calibers and higher NHF flow rates.

In conclusion, our data show that neither NHF at 25 L/min administered through medium-sized nasal prongs, nor ERL significantly improved the regional lung ventilation of patients with stable COPD with gas trapping, assessed based on the registration of expiratory and inspiratory CT images. Further study including a larger number of subjects is needed to investigate whether NHF improves regional lung function at higher flow rates.

## Data Availability Statement

The raw data supporting the conclusions of this article will be made available by the authors, without undue reservation.

## Ethics Statement

The studies involving human participants were reviewed and approved by Comité de Protection des Personnes, Nord Ouest. The patients/participants provided their written informed consent to participate in this study.

## Author Contributions

JC, RT, J-LP, GF, and SB conceived the study. JC, GF, and SB performed image acquisition. JC, LB, MM, and SB analyzed image data. JC and SB performed statistical analysis. JC, RT, J-LP, GF, and SB interpreted the findings. SB drafted the manuscript. All authors have seen and edited the submitted manuscript.

## Conflict of Interest

J-LP has received grants and research funds for other studies from: Air Liquide Foundation, Agiradom, AstraZeneca, Fisher & Paykel, Mutualia, Philips, Resmed, Vitalaire, Boehringer Ingelheim, Jazz Pharmaceuticals, Night Balance, and Sefam. SB received funding from Fisher & Paykel for this study. The funder was involved in the study design, but not the collection, analysis, interpretation of data, the writing of this article, or the decision to submit it for publication. The remaining authors declare that the research was conducted in the absence of any commercial or financial relationships that could be construed as a potential conflict of interest.
