# Supplementary material for: Nasal High Flow at 25 L/min or Expiratory Resistive Load Do Not Improve Regional Lung Function in Patients With COPD: A Functional CT Imaging Study
Source: Front Physiol. 2021 Jun 10;12:683316. doi: 10.3389/fphys.2021.683316 (PMC8222991; doi:10.3389/fphys.2021.683316)
Supplement: Supplementary file 1 [file Data_Sheet_1.docx]

Supplementary Material

# Supplementary methods

Images were processed with the python programming language (Python Software Foundation; Python Language Reference, version 2.7), running on a desktop computer (CPU: Intel Xenon @2.4 GHz x 16, 126 GB of RAM and NVIDIA Quadro K5000 GPU). Segmentation of the aerated lung tissue from the CT images was performed using a iterative region growing algorithm(1). Within the segmented lung, an aerated voxel was defined by a lung-tissue density lower than the median of the distribution plus two standard deviations: μ+2σ. An elastic 3D registration method was used to compute motion and deformation between inspiratory and expiratory lung images(3, 4). This method was based on minimization of the Sum of Squared Differences (SSD) between the elastically transformed expiratory image and his static inspiratory counterpart, defined as:

$$SSD= \frac{1}{\left\| \left. I \right\| \right.} \sum_{\left( x,y,z \right)\in I} {(I_{T}(g(x,y,z))-I_{R}(x,y,z))}^{2}$$

Where *x, y ,z* are the 3D coordinates in the set of all voxel coordinates *I*; *I_T_* is the test image (expiratory phase) and *I_R_* the reference image (inspiratory phase); *g* the deformation function*.* The iterative correction of the registered image was performed by B-Spline transformation and B-Spline interpolation(3). All registration calculations were performed with the Insight Segmentation and Registration Toolkit library (ITK)(2).

# References

1. **Adams R, and Bischof L**. Seeded region growing. *Pattern Analysis and Machine Intelligence, IEEE Transactions on* 16: 641-647, 1994.

2. **Ibanez L, Schroeder W, Ng L, and Cates J**. The ITK software guide. 2003.

3. **Kybic J, and Unser M**. Fast parametric elastic image registration. *IEEE transactions on image processing* 12: 1427-1442, 2003.

4. **Yoo TS, Ackerman MJ, Lorensen WE, Schroeder W, Chalana V, Aylward S, Metaxas D, and Whitaker R**. Engineering and algorithm design for an image processing Api: a technical report on ITK--the Insight Toolkit. *Stud Health Technol Inform* 85: 586-592, 2002.

**
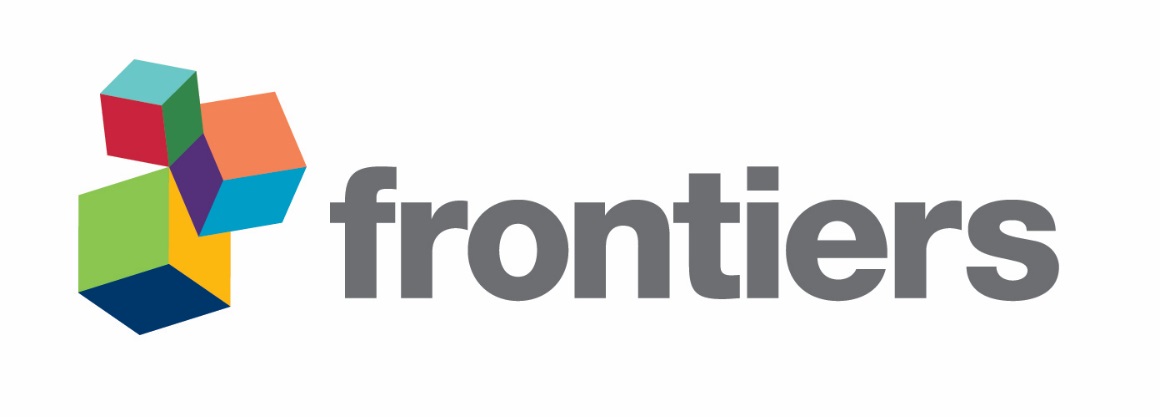
**
